# The Race for ACE: Targeting Angiotensin-Converting Enzymes (ACE) in SARS-CoV-2 Infection

**DOI:** 10.1155/2022/2549063

**Published:** 2022-05-27

**Authors:** Elisabeth Schieffer, Bernhard Schieffer

**Affiliations:** Department of Cardiology, Angiology and Critical Care Medicine, Philipps University, Marburg, Germany

## Abstract

The SARS-CoV-2 virus is spreading around the world, and its clinical manifestation COVID-19 is challenging medical, economic, and social systems. With more and more scientific and social media reports on the COVID-19 pandemic appearing, differences in geographical presentations and clinical management occur. Since ACE2 (angiotensin-converting enzyme 2) is the gatekeeper receptor for the SARS-CoV-2 virus in the upper bronchial system, we here focus on the central role of the renin-angiotensin aldosterone system (RAAS) in the SARS-CoV-2 virus infection, the role of pharmacological RAAS inhibitors, and specific genetic aspects, i.e., single nucleotide polymorphisms (SNP) for the clinical outcome of COVID-19. We aimed to bring together clinical, epidemiological, molecular, and pathophysiological and pharmacological data/observations on cardiovascular aspects in the actual SARS-CoV-2 virus pandemic. In detail, we will report controversies about the Yin-Yan between ACE2 and ACE1 and potential implications for the treatment of hypertension, coronary artery disease, and heart failure. Here, we summarize the encouraging and dynamic global effort of multiple biomedical disciplines resulted in astonishing fight against COVID-19 targeting the renin-angiotensin-aldosterone system, yet the race for ACE just begun.

## 1. Introduction

In early 2020, reports from China and Italy showed that genetic predispositions and individual comorbidities like hypertension, diabetes, or obesity are more prevalent in COVID-19 patients, a novel disease entity caused by the SARS-CoV-2 virus [1, 2]. This virus enters human cells through the ACE2 enzyme (angiotensin I-converting enzyme II) which is a critical part of the renin-angiotensin aldosterone system (RAAS), and imbalance of this system has been reported in hypertension and other cardiometabolic pathologies [3–5]. Therefore, we here take a closer look at the RAAS and the mimicry of its intrinsic feedback loops and compensatory mechanisms including the kallikrein-kinin system. In general, the RAAS elicits its effects via two pathways. (1) The canonical angiotensin I-converting enzyme 1 (ACE1), angiotensin II (Ang II), and angiotensin II receptor type 1 (AT1R) axis can be pharmacologically modulated by either ACE1-inhibitors or AT1R-blocker (ARB) at the cell membrane level.^6^ The other one is the angiotensin I-converting enzyme II (ACE2) pathway transforming angiotensin (1–7) (Ang (1–7)) as an agonist of Mas receptor (ACE2/Ang (1–7)/MAS pathway). The Yin-Yan balance between these two systems is crucial for the development of lung injury and has been described in older age as well as in hypertension [6, 7]. The complexity of the RAAS system is further aggravated since ACE2 is a regulating peptidase for the kallikrein-kinin system controlling the stimulation of the bradykinin B1 and B2 receptors [8, 9].

Beside this enzyme mimicry, gender differences were also described for RAAS-dependent blood pressure regulation and kidney function. In males, the ACE/Ang II/AT1R pathway is enhanced, whereas in premenopausal females, the balance is shifted towards the more protective ACE2/Ang (1–7)/MasR and AT2 receptor pathways [10]. In line with this finding, premenopausal women are less likely to have hypertension as compared to aged-matched men. In addition, for ACE2, different polymorphisms are described as well as SNPs within the ACE2 gene that genetical and epigenetical factors might influence the balance of RAAS. Thus, since the majority of COVID-19 patients are of older age with hypertension and diabetes as comorbidities, it is worth speculating that RAAS modulation, i.e., by ARB's, has significant clinical impact on SARS-CoV-2 virus infections. The question however remains whether we do have any evidence for this latter.

## 2. RAAS and SARS-CoV-2

Twenty years ago (2002-2003), the first SARS coronavirus pandemic killed hundreds of citizens in China and Southeast Asia. At that time, ACE2 was identified as a crucial SARS virus receptor in vitro and in vivo [11]. Now, since March 2020, we were facing the next coronavirus (SARS-CoV-2) threat with its disease named COVID-19 and the fact that ACE2 plays a major role in SARS-CoV-2 infections in the past brought different researchers together formerly working in the field of hypertension or virology. The focus is again on the ACE2-dependent ANG1-7-MAS axis, which has been shown to be protective in lung failure [12]. More recently, the SARS-CoV-2, the cause of severe acute respiratory syndrome (SARS), utilizes ACE2 as an essential receptor for cell fusion and in vivo infections [13]. In this regard, recent evidence occurred that ACE2 protects murine lungs from acute lung injury as well as SARS-spike protein-mediated lung injury, suggesting a dual role of ACE2 in SARS-CoV-2 infections and protection from acute respiratory lung injury (ARDS). Since every information is desperately needed to optimize our actual virus-defense strategy beyond quarantine/containment, the potential impact of ACE and COVID-19 is discussed. An encouraging global effort of multiple biomedical disciplines resulted in astonishing scientific fight against COVID-19 not only in the Chinese population of Han and non-Han citizens of Wuhan but also in the US and European countries. Recent clinical data suggest that a mechanism of lung injury during the viral infection may be through inappropriate effects of excess circulating angiotensin II protein [14], which is floating around since ACE2 that would normally be soaking it up is occupied by coronavirus particles. If that is the problem, then increasing the amount of ACE2 protein might be paradoxically but just what we want to do in order to restore some balance to the angiotensin system. In that case, administering more angiotensin receptor antagonists would be an effective way to upregulate the production of ACE2. In fact, the first clinical trial [15] investigating this question by using low-dose losartan (2 × 25 mg) was underpowered with only 60 patients and showed discouraging results. Nevertheless, we recently summarized that ARB significantly differ with their AT1-receptor binding activity, their active metabolites (i.e., cyclooxygenase 2 and thromboxane inhibition), and liposolubility which might be responsible for a potential beneficial clinical effect in larger clinical trials [16]. Thus, we are convinced that the critical role of the RAAS in COVID-19 is not completely understood, but increasing amount of data regarding the impact of SARS-CoV-2 infection on the RAAS and kallikrein-kinin system bringing more pieces to the puzzle and imply that the race for ACE has just began.

## 3. The Hypothesis

We are currently (March 2022) realizing that we are unable to prevent SARS-CoV-2 virus infestation of our citizens, but can we mitigate the clinical presentation of COVID-19 patients and thereby reduce mortality rates? In detail, should we continue blocking the RAAS in COVID-19 patients and is one RAAS blockade better than the other (ACE-inhibition versus AT1R-blockade)?

## 4. Genetical Insights

DNA variations within the genes of ACE1 and ACE2 have been shown to be involved in the etiology of several common diseases and their therapeutic response [17, 18]. ACE2 is similar to ACE1 in that it is a membrane-associated and secreted enzyme that is predominantly expressed in the endothelium. ACE2 is highly expressed in the kidney, testis, and heart and has been implicated in hypertension and hyperlipidemia [19]. At least 12 peptides that are significantly hydrolyzed by ACE2 have been identified, including angiotensin I and II [20]. In contrast to ACE1, ACE2 is located on chromosome Xp22. Due to this, both genetic and epigenetic effects may influence ACE2 function, because males are hemizygotes at the locus, and females show mosaic imprinting of the gene due to X-chromosome inactivation. Thus, gender may explain potential differences in clinical outcome, a latter which is supported by recent data from Italy reporting significant differences in mortality (70% male versus 33% female casualties) in COVID-19 [21]. Moreover, this observation may not only explain potential differences in outcome in male versus female patients but also shed light on prevalence of infection rates. Additional genetic aspects are “traditional” polymorphisms for ACE1 which have been investigated for decades [22]. That is, the human ACE1 gene on chromosome 17q23 consists of 26 exons and contains an insertion (I) or deletion (D) polymorphism of 287 bp in intron 16. Although the functional consequences of this genetic variation are still poorly understood, associations of this polymorphism with the development of COPD, pneumonia, myocardial infarction, and deterioration of kidney function in diabetes mellitus have been suggested in multiple populations [23, 24]. In 1990, Rigat et al. showed that the DD genotype is associated with increased cellular and circulating levels of ACE, which can worsen the long-term course in patients with COPD [25, 26]. Itoyama et al. studied 197 subjects, with 44 SARS patients, 103 healthy subjects being in contact with SARS patients, and 50 healthy controls without any SARS contact [27]. They were able to identify subjects with the homozygote ACE DD polymorphism to be at significantly greater risk for a severe, hypoxemic course of the infection. By this, they conclude that ACE1 might be one of the candidate genes that influence the progression of SARS pneumonia. Although the ID polymorphism differentiates the 2 clades of the phylogenetic tree and SNPs within the ACE gene, contradicting results from association studies regarding ACE levels have been reported. This might be related to the 50/50 distribution of both alleles in different populations. For this purpose, it is more accurate for future studies to examine additional SNPs within the absolute linkage disequilibrium with the commonly typed Alu insertion/deletion polymorphism [28, 29].

**Figure 1:**
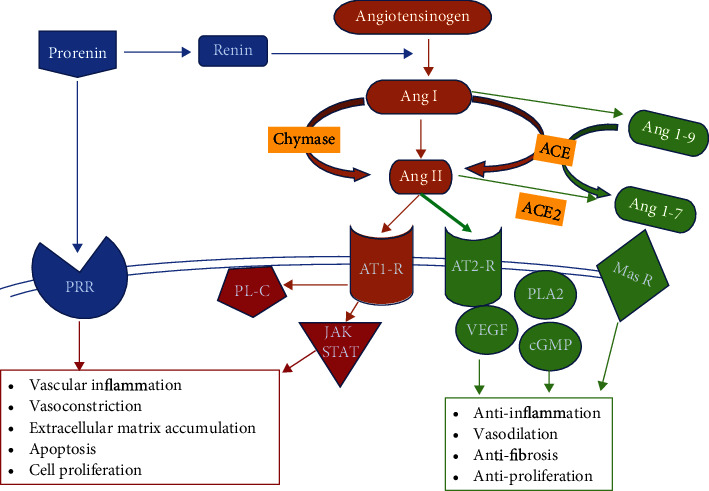
Graphical illustration summarizing the canonical renin-angiotensin system cascade. In brief, prorenin is released by the kidney and activates via its prorenin-receptor proinflammatory pathways. Angiotensinogen as acute phase protein released from the liver is cleaved by renin to angiotensin I which is cleaved to the octapeptide angiotensin II (ANG II). In mast cells, this process uses an alternative pathway using the mast-cell chymase (orange). ANG II then activates via its G-protein coupled AT1-receptor proinflammatory pathways by stimulating the JAK-Stat signaling cascade as well as the phospholipase gamma signaling cascade (red). By stimulating its AT2-receptor, ANG II promotes the activation of predominantly anti-inflammatory pathways, i.e., cGMP, phospholipase A, or VEGF. ACE2 degrades ANG II to angiotensin 1-7 which in turn also stimulates regenerative pathways via the Mas receptor.

Kuba et al. showed a decrease in ACE2 expression once ACE2 was occupied by SARS-CoV [11]. One might speculate a negative feedback mechanism of a nonfunctional virus depressed ACE2 protein. However, a decrease in ACE2 and/or a virus-induced downregulation of ACE2 activity (due to virus-mediated internalization of ACE2) leads to an increase of angiotensin II with all deleterious effects. Nevertheless, Kuba et al. showed that ACE2 knockout mice were protected against SARS-CoV- induced lung damage. In addition, Kuba et al. attenuated lung failure by blocking the renin-angiotensin pathway with high doses of the angiotensin II type 1 receptor antagonist (AT1R antagonist) losartan (15 mg/kg) in a mouse model, suggesting a therapeutical tool for COVID-19 patients [11]. That is, ACE1 cleaves angiotensin I to angiotensin II but in a downstream step, ACE2 inactivates the potentially lung damaging angiotensin II (see Figure 1). The exciting work by Kuba et al. underlined the crucial role of ACE2 as SARS-CoV-2 virus receptor in vivo; that is, SARS-CoV-2 binds to ACE2 and is internalized. This in turn leads to a downregulation of ACE2 which subsequently promotes excessive angiotensin II production by the related ACE1. In this regard, the ACE-DD genotype contributes to this scenario by being associated with enhanced circulating and tissue ACE1 concentration and activity and ACE I/D polymorphism with pulmonary embolism in COVID-19 [30]. Decreased ACE2 level in contrast leads to reduced heptapeptide angiotensin 1-7 that has strong vasodilatory function and serves as a negative regulator of angiotensin II effects. There are polymorphisms in the genome, such as insertion-deletion modifications for ACE2. Levels of ACE2 across humans will vary according to inherent genomic expression [29]. Taken together, our hypothesis could be that the severity of response to SARS-CoV-2 virus infection might correlate to factors involving ACE2 gene expression levels and/or gene polymorphisms. Whether this latter accounts (at least in part) for the exaggerated severity of COVID-19 remains to be elucidated. However, first results regarding genotype analysis revealed contradictory results. Möhlendick et al. found ACE2 GG-genotype correlating with COVID-19 disease severity, but no correlation with ACE polymorphism [31]. In contrast, Gómez et al. found a weak significance between ACE D/D genotype and severe COVID-19 in association with hypertension, but no influence of ACE2 polymorphim [32].

## 5. Crosstalk between the RAAS and the Kallikrein/Kinin System

The kallikrein-kinin system is viewed as a natural counterbalance to the RAAS due to blood pressure decreasing effects via bradykinin, which is converted from kininogens [33]. Kinins bind to bradykinin B1 or B2 receptors (B1R and B2R). B2R is expressed ubiquitously, and B1R is generally absent in healthy tissue but expressed during inflammation. Receptor activation can result in vasodilation, capillary permeability, nociceptor sensitization and pain, mediation protective, or deleterious effects [34]. Besides binding of bradykinin to B2R, it can be degraded by angiotensin-converting enzyme or be converted to des-arg9-bradykinin (DABK). DABK binds to B1-receptor (B1R) and seems to accentuate an inflammation cascade [8]. DABK can be degraded by ACE2, resulting in less inflammation. Since SARS-CoV-2 infection results in ACE2 internalization, this might result in an imbalance within the kallikrein-kinin system shifting the balance toward the activation of DABK/B1-receptor with the clinical effects of hyperinflammation and local vascular leakage [35].

The kallikrein-kinin system can also activate the coagulation system via factor XII inducing a procoagulatory state [36]. Consequently, clinical approaches targeting solely the RAAS system but not the kallikrein-kinin system might not be effective in limiting the state of hyperinflammation and procoagulation as typical clinical presentation seen in severe advanced SARS-CoV-2 infections as well as in long COVID-19 patients.[37, 38]

Thus, the race for ACE is a search for drugs that could target both the RAAS and kallikrein-bradykinin system in order to reconstitute the balance within these closely linked pathways, thereby subsequently fighting successfully COVID-19 and thereby potentially preventing long COVID-19.

Due to their anti-inflammatory effects, studies investigating ARB in COVID-19 have been performed. Puskarich et al. could not find an effect of losartan in outpatients with mild COVID-19 [15], whereas in an open multicenter randomized trial, telmisartan resulted in decreased morbidity and mortality [39]. As suggested by Rothlin et al., different receptor binding affinities or lipophilic properties of ARB might impact clinical outcome and should be further evaluated [40]. In addition, since ARB upregulates ACE-2, which can degrade DABK, the agonist of the B1R, this might decease the inflammatory response as well.

However, this higher ACE2 level will also help shedding circulating angiotensin II, thereby protecting from pulmonary hypertension and also block angiotensin II-AT1-receptor mediated interleukin 6 release (see also Figure 1) [41–43]. This hypothesis is supported by Dimitrov who reported that SARS-CoV-2 targets ACE2 as the receptor binding domain for its S-protein [44]. But can we conclude that angiotensin II type 1 receptor blockers (ARBs) are more beneficial than ACE-inhibitors for patients infected by SARS-CoV-2 who suffer from pulmonary failure? The actual SARS-CoV-2 virus strain shares 72% amino acid sequence identity with the 2003 SARS coronavirus strain [45]. Thus, we assume that both viruses elicit identical effects in vivo. If so, application of an ARBs like losartan that enhances circulating ACE1 and ACE2 activities might mitigate SARS virus infection by modulating angiotensin II-stimulated IL-6 induced inflammation, and reducing circulating angiotensin II levels and enabling more AT2-receptor stimulation [2, 17].∗ Nevertheless, angiotensin II-AT1-receptor blockade will not prevent virus invasion.

## 6. Clinical Conclusion

COVID-19 disease was announced pandemic in late 2020. Since ACE2 is crucial for the virus entry to the host, clinical efforts should focus on targeting the ACE-system. Older age people and those with cardiovascular comorbidities (60-75%) are at higher risk for an imbalance between the ACE1-AT1R and the ACE2-MAS axis [25].

In a first step, we should focus on the collection of clinical results to determine whether differences occur in the clinical outcome of SARS-CoV-2-infected patients on ACE-inhibitors or ARBs or none of both. Furthermore, prospective analysis should evaluate whether polymorphisms of ACE1 and ACE2 might affect the clinical outcome of COVID-19 patients and if not whether additional SNP analysis of the ACE1 and ACE2 are required. However, the individual tissue-specific expression and activity of both ACE-enzymes and their Yin-Yan interplay may predict the disease severity and chronification following SARS-CoV-2 virus infection. Moreover, pulmonary edema is a prominent symptom of severely infected patients. Whether blockade of the RAAS system by using ARB's elicits additional effects on top of standard therapy will be determined in future trials.

As discussed here, ARB might be of special interest in COVID-19, since they can not only decrease the inflammatory pathway directly but also increase ACE2. The latter one can block DABK, the agonist of B1R, thus decreasing inflammation via another pathway. Future studies should address AT1R-blocker in COVID-19 and long COVID-19 in more detail. In addition, prospective analysis regarding the impact of ACE/ACE2 polymorphisms or SNP analysis affecting the clinical outcome of COVID-19 patients might lighten the impact of RAAS in the context of COVID-19. Thus, the race for ACE is ongoing, challenging researchers and clinicians not only as specialists but also as interdisziplinary teams.

## 7. Critical Comments

The moment we wrote this review, COVID-19 pandemic demonstrates in a disillusioning manner that this tricky virus by targeting the ACE2 enzyme as gate keeper in the bronchial system bypasses the human immune defense system and leaves us with a tremendous amount of formerly healthy people with long COVID-19 disease for the next decades. In this regard, it remains puzzling that gene polymorphisms of the ACE1 and ACE2 genes may contribute to such a devastating clinical effect. However, a phase I trial targeting ACE2 by applying a soluble human ACE2 protein (rhACE2) to treat severely infected patients with mechanical ventilation has just launched (Apeiron). However, beyond established risk factors such as age, diabetes, or hypertension, we might now focus on additional individual biomarkers as well. As described above, there is some evidence that pretreatment with ACE-inhibitors or ARB as well as the individual genetic background might be beneficial for the clinical outcome in COVID-19 disease. Nowadays, innovative app-based technologies and artificial intelligence can help to rapidly collect and analyze data to answer immediately the above posed questions. This might help to stratify the individual risk and the demand of regional medical care and thereby hopefully shed further light into the role of the RAAS in SARS-CoV-2 virus infection.

## Abbreviations

RAAS: Renin-angiotensin aldosterone system
ACE: Angiotensin-converting enzyme
AT1: Angiotensin II type 1 receptor
SARS-CoV-2 virus: COVID-19 disease.

## References

[1] Chen N., Zhou M., Dong X. (2020). Epidemiological and clinical characteristics of 99 cases of 2019 novel coronavirus pneumonia in Wuhan, China: a descriptive study. *Lancet*.

[2] Grasselli G., Zangrillo A., Zanella A. (2020). Baseline characteristics and outcomes of 1591 patients infected with SARS-CoV-2 admitted to ICUs of the Lombardy Region, Italy. *Journal of the American Medical Associatio*.

[3] Unger T. (2002). The role of the renin-angiotensin system in the development of cardiovascular disease. *The American Journal of Cardiology*.

[4] Schmieder R. E., Hilgers K. F., Schlaich M. P., Schmidt B. M. (2007). Renin-angiotensin system and cardiovascular risk. *The Lancet*.

[5] Ferrario C. M., Strawn W. B. (2006). Role of the renin-angiotensin-aldosterone system and proinflammatory mediators in cardiovascular disease. *The American Journal of Cardiology*.

[6] Te Riet L., van Esch J. H. M., Roks A. J. M., van den Meiracker A. H., Danser A. H. J. (2015). Hypertension. *Circulation Research*.

[7] Zhang P., Zhu L., Cai J. (2020). Association of inpatient use of angiotensin converting enzyme inhibitors and angiotensin II receptor blockers with mortality among patients with hypertension hospitalized with COVID-19. *Circulation Research*.

[8] Bekassy Z., Lopatko Fagerström I., Bader M., Karpman D. (2021). Crosstalk between the renin-angiotensin, complement and kallikrein-kinin systems in inflammation. *Nature Reviews. Immunology*.

[9] Patel S., Rauf A., Khan H., Abu-Izneid T. (2017). Renin-angiotensin-aldosterone (RAAS): the ubiquitous system for homeostasis and pathologies. *Biomedicine & Pharmacotherapy*.

[10] Hilliard L. M., Sampson A. K., Brown R. D., Denton K. M. (2013). The ‘his and hers’ of the renin-angiotensin system. *Current Hypertension Reports*.

[11] Kuba K., Imai Y., Rao S. (2005). A crucial role of angiotensin converting enzyme 2 (ACE2) in SARS coronavirus-induced lung injury. *Nature Medicine*.

[12] Supé S., Kohse F., Gembardt F., Kuebler W. M., Walther T. (2016). Therapeutic time window for angiotensin-(1–7) in acute lung injury. *British Journal of Pharmacology*.

[13] Xing W., Hejblum G., Leung G. M., Valleron A.-J. (2010). Anatomy of the epidemiological literature on the 2003 SARS outbreaks in Hong Kong and Toronto: a time-stratified review. *PLoS Medicine*.

[14] Liu Y., Yang Y., Zhang C. (2020). Clinical and biochemical indexes from 2019-nCoV infected patients linked to viral loads and lung injury. *Science China. Life Sciences*.

[15] Puskarich M. A., Cummins N. W., Ingraham N. E. (2021). A multi-center phase II randomized clinical trial of losartan on symptomatic outpatients with COVID-19. *EClinicalMedicine*.

[16] Schmidt B., Schieffer B. (2003). Angiotensin II AT1 receptor antagonists. Clinical implications of active metabolites. *Journal of Medicinal Chemistry*.

[17] Mizuiri S., Ohashi Y. (2015). ACE and ACE2 in kidney disease. *World J. Nephrol.*.

[18] Calabrese C., Annunziata A., Coppola A. (2021). ACE gene I/D polymorphism and acute pulmonary embolism in COVID19 pneumonia: a potential predisposing role. *Frontiers in Medicine*.

[19] Pan Y., Wang T., Li Y. (2018). Association of ACE2 polymorphisms with susceptibility to essential hypertension and dyslipidemia in Xinjiang, China. *Lipids in Health and Disease*.

[20] Vickers C., Hales P., Kaushik V. (2002). Hydrolysis of biological peptides by human angiotensin-converting enzyme- related carboxypeptidase. *The Journal of Biological Chemistry*.

[21] https://globalhealth5050.org/the-sex-gender-and-covid-19-project/.

[22] Pinheiro D. S., Santos R. S., Jardim P. C. B. V. (2019). The combination of ACE I/D and ACE2 G8790A polymorphisms revels susceptibility to hypertension: a genetic association study in Brazilian patients. *PLoS One*.

[23] Ma Y., Tong X., Liu Y., Liu S., Xiong H., Fan H. (2018). ACE gene polymorphism is associated with COPD and COPD with pulmonary hypertension: a meta-analysis. *International Journal of Chronic Obstructive Pulmonary Disease*.

[24] Baudin B. (2002). New aspects on angiotensin-converting enzyme: from gene to disease. *Clinical Chemistry and Laboratory Medicine*.

[25] Rigat B., Hubert C., Alhenc-Gelas F., Cambien F., Corvol P., Soubrier F. (1990). An insertion/deletion polymorphism in the angiotensin I-converting enzyme gene accounting for half the variance of serum enzyme levels. *The Journal of Clinical Investigation*.

[26] Forth R., Montgomery H. (2003). ACE in COPD: a therapeutic target?. *Thorax*.

[27] Itoyama S., Keicho N., Quy T. (2004). ACE1 polymorphism and progression of SARS. *Biochemical and Biophysical Research Communications*.

[28] Rieder M. J., Taylor S. L., Clark A. G., Nickerson D. A. (1999). Sequence variation in the human angiotensin converting enzyme. *Nature Genetics*.

[29] Zhu X., McKenzie C. A., Forrester T. (2000). Localization of a small genomic region associated with elevated ACE. *American Journal of Human Genetics*.

[30] Verma S., Abbas M., Verma S. (2021). Impact of I/D polymorphism of angiotensin-converting enzyme 1 (ACE1) gene on the severity of COVID-19 patients. *Infection, Genetics and Evolution*.

[31] Möhlendick B., Schönfelder K., Breuckmann K. (2021). ACE2 polymorphism and susceptibility for SARS-CoV-2 infection and severity of COVID-19. *Pharmacogenetics and Genomics*.

[32] Gómez J., Albaiceta G. M., García-Clemente M. (2020). Angiotensin-converting enzymes (ACE, ACE2) gene variants and COVID-19 outcome. *Gene*.

[33] Hamid S., Rhaleb I. A., Kassem K. M., Rhaleb N.-E. (2020). Role of Kinins in hypertension and heart failure. *Pharmaceuticals*.

[34] Lau J., Rousseau J., Kwon D., Bénard F., Lin K.-S. (2020). A systematic review of molecular imaging agents targeting bradykinin B1 and B2 receptors. *Pharmaceuticals*.

[35] Ramos S. G., da Cruz Rattis B. A., Ottaviani G., Celes M. R. N., Dias E. P. (2021). ACE2 down-regulation may act as a transient molecular disease causing RAAS dysregulation and tissue damage in the microcirculatory environment among COVID-19 patients. *The American Journal of Pathology*.

[36] Maas C., Renné T. (2018). Coagulation factor XII in thrombosis and inflammation. *Blood*.

[37] Silva Andrade B., Siqueira S., de Assis Soares W. R. (2021). Long-COVID and post-COVID health complications: an up-to-date review on clinical conditions and their possible molecular mechanisms. *Viruses*.

[38] Asakura H., Ogawa H. (2021). COVID-19-associated coagulopathy and disseminated intravascular coagulation. *International Journal of Hematology*.

[39] Duarte M., Pelorosso F., Nicolosi L. N. (2021). Telmisartan for treatment of Covid-19 patients: an open multicenter randomized clinical trial. *EClinicalMedicine*.

[40] Rothlin R. P., Vetulli H. M., Duarte M., Pelorosso F. G. (2020). Telmisartan as tentative angiotensin receptor blocker therapeutic for COVID-19. *Drug Development Research*.

[41] Gurwitz D. (2020). Angiotensin receptor blockers as tentative SARS-CoV-2 therapeutics. *Drug Development Research*.

[42] Sun Z., Thilakavathy K., Kumar S. S., He G., Liu S. V. (2020). Potential factors influencing repeated SARS outbreaks in China. *International Journal of Environmental Research and Public Health*.

[43] Kickbusch I., Leung G. (2020). Response to the emerging novel coronavirus outbreak. *BMJ*.

[44] Dimitrov D. S. (2003). The secret life of ACE2 as a receptor for the SARS virus. *Cell*.

[45] Chen Y., Guo Y., Pan Y., Zhao Z. J. (2020). Structure analysis of the receptor binding of 2019-nCoV. *Biochemical and Biophysical Research Communications*.

